# Challenges Faced by Nursing Home Direct Care Workers During the COVID-19 Pandemic: A Comparison Across Care Settings

**DOI:** 10.1093/geroni/igab046.955

**Published:** 2021-12-17

**Authors:** Natasha Bryant, Verena Cimarolli, Robyn Stone

**Affiliations:** 1 LeadingAge LTSS Center @UMass Boston, Washington, District of Columbia, United States; 2 LeadingAge, Washington, District of Columbia, United States

## Abstract

The COVID-19 pandemic has generated awareness of the value of the direct care workforce to provide care in settings serving those most at risk from the disease. However, few studies have gauged the impact of COVID-19 on this workforce and their pandemic-related challenges. The purpose of this study was to examine the challenges and stress experienced by direct care workers (N=1,414) and their perceptions of preparation and quality of employer communication during this health crisis. Nursing home (NH) workers reported separation from family members and understaffing as the top external and work-related challenges. They felt adequately prepared and gave their employers high marks for communicating with them during the pandemic. NH direct care workers were more likely to report increased workload and understaffing as a challenge compared to workers in home and community-based settings. They also experienced a significantly higher number of work-related challenges compared to workers in assisted living.

